# Human papillomavirus mediated inhibition of DNA damage sensing and repair drives skin carcinogenesis

**DOI:** 10.1186/s12943-015-0453-7

**Published:** 2015-10-29

**Authors:** Martin Hufbauer, James Cooke, Gijsbertus T. J. van der Horst, Herbert Pfister, Alan Storey, Baki Akgül

**Affiliations:** Institute of Virology, University of Cologne, Fürst-Pückler-Str. 56, Cologne, 50935 Germany; Centre for Cutaneous Research, The Blizard Institute, Barts and The London School of Medicine and Dentistry, Queen Mary University of London, London, E1 2AT UK; MGC, Department of Genetics, Center for Biomedical Genetics, Erasmus University Medical Center, Rotterdam, 3000 CA The Netherlands; Department of Oncology, Weatherall Institute of Molecular Medicine, University of Oxford, Oxford, OX3 9DS UK

**Keywords:** DNA damage repair, Human papillomavirus 8 (HPV8), HPV of genus betapapillomavirus, E6 oncoprotein, Non-melanoma skin cancer, Photolyase

## Abstract

**Background:**

The failure to mount an effective DNA damage response to repair UV induced cyclobutane pyrimidine dimers (CPDs) results in an increased propensity to develop cutaneous squamous cell carcinoma (cSCC). High-risk patient groups, such as organ transplant recipients (OTRs) frequently exhibit field cancerization at UV exposed body sites from which multiple human papillomavirus (HPV)-associated cSCCs develop rapidly, leading to profound morbidity and increased mortality. In vitro molecular evidence indicates that HPV of genus beta-papillomavirus (β-PV) play an important role in accelerating the early stages of skin tumorigenesis.

**Methods:**

We investigated the effects of UV induced DNA damage in murine models of β-PV E6 oncoprotein driven skin tumorigenesis by crossing K14-HPV8-E6wt mice (developing skin tumors after UV treatment) with K14-CPD-photolyase animals and by generating the K14-HPV8-E6-K136N mutant mouse strain. Thymine dimers (marker for CPDs) and γH2AX (a marker for DNA double strand breaks) levels were determined in the murine skin and organotypic skin cultures of E6 expressing primary human keratinocytes after UV-irradiation by immunohistochemistry and in cell lines by In Cell Western blotting. Phosphorylation of ATR/Chk1 and ATM were assessed in cell lines and organotypic skin cultures by Western blots and immunohistochemistry.

**Results:**

Skin tumor development after UV-irradiation in K14-HPV8-E6wt mice could completely be blocked through expression of CPD-photolyase. Through quantification of thymine dimers after UV irradiation in cells expressing E6 proteins with point mutations at conserved residues we identified a critical lysine in the C-terminal part of the protein for prevention of DNA damage repair and p300 binding. Whereas all K14-HPV8-E6wt animals develop skin tumors after UV expression of the HPV8-E6-K136N mutant significantly blocked skin tumor development after UV treatment. The persistence of CPDs in hyperproliferative epidermis K14-HPV8-E6wt skin resulted in the accumulation of γH2AX foci. DNA damage sensing was impaired in E6 positive cells grown as monolayer culture and in organotypic cultures, due to lack of phosphorylation of ATM, ATR and Chk1.

**Conclusion:**

We showed that cells expressing E6 fail to sense and mount an effective response to repair UV-induced DNA lesions and demonstrated a physiological relevance of E6-mediated inhibition of DNA damage repair for tumor initiation. These are the first mechanistical in vivo data on the tumorigenicity of HPV8 and demonstrate that the impairment of DNA damage repair pathways by the viral E6 protein is a critical factor in HPV-driven skin carcinogenesis.

**Electronic supplementary material:**

The online version of this article (doi:10.1186/s12943-015-0453-7) contains supplementary material, which is available to authorized users.

## Background

Exposure to solar ultraviolet B wavelengths (UVB) is the principal risk factor for skin cancer development. UVB damages DNA through the formation of the potentially mutagenic photoproducts, cyclobutane-pyrimidine-dimers (CPDs) or 6–4-photoproducts (6-4PPs) [1]. Transcriptome analysis showed that the most prominent pathway induced by CPDs was associated with DNA double-strand break (DSB). These results implied that the conversion of unrepaired CPDs into DSB during DNA replication constitutes the principal source of UV-mediated cytotoxicity [2] and that CPDs are the principal lesions accounting for most DNA damage-dependent biological effects [3]. The importance of DNA repair mechanisms in preventing skin cancer development is demonstrated clearly in certain human syndromes such as xeroderma pigmentosum (XP), characterized as being defective in DNA repair processes that exacerbate the clinical effects of unrepaired DNA damage resulting in genetic instability. XP is characterized by severe UV sensitivity resulting in a 10,000-fold increased risk for skin cancer development on UV-exposed tissues [4] indicating a clear causative relationship between unrepaired DNA lesions and cancer. Experiments using transgenic mice expressing *Potorous tridactylus* CPD-photolyase under the control of the keratin-14 promoter (K14-CPD-PL) indicated that fast removal of CPDs from K14-permissive cells dramatically decreased the incidence of skin cancer in UV-treated animals [5–7].

In addition to UV-radiation, there is an emerging pathogenic role for human papillomavirus (HPV) of genus betapapillomavirus (β-PV, e.g. HPV5 and HPV8) in the initiation phase of cutaneous SCC (cSCC) in iatrogenically immunosuppressed patients [8–10]. These aggressive tumors appear on sun-exposed areas of skin that exhibit’field cancerization’, with concomitant increased morbidity through development of multiple tumors that develop rapidly.

The carcinogenic capacity of HPV8 could be demonstrated in transgenic mice, expressing the complete early gene region (CER) of HPV8 under control of the human keratin-14 promoter. These K14-HPV8-CER mice spontaneously developed papillomas, characterized by varying degrees of epidermal dysplasia and SCC without any treatment with physical or chemical carcinogens within few months [11]. Transgenic mice expressing only the HPV8 E6 gene from the K14 promoter (K14-HPV8-E6) also showed a high penetrance of papillomatosis, followed by progression to dysplasia and SCC. A single UVA/B irradiation of K14-HPV8-E6 transgenic mice accelerated tumor development as papillomas arose within three weeks after treatment [12]. Papilloma development was always preceded by an increased transgene expression and knock-down of E6 mRNA by HPV8-E6-specific siRNA in the context of CER led to a delay and a lower incidence of papilloma development. This indicated that E6 is the major oncogene of HPV8 in the murine epidermis, since early increase of E6 expression is necessary and sufficient for induction of papilloma formation [13]. In vitro studies revealed that inhibition of UV-induced apoptosis [14, 15] and DNA damage repair after UV-irradiation (reviewed in [16]) represent two activities of the β-PV E6 protein, which may result in survival of damaged cells.

At present however, the different contributions of the inhibition of apoptosis and interference with DNA repair in HPV E6-driven tumorigenesis are yet to be resolved. In this study we provide compelling evidence that interference with the DNA repair pathway is necessary and obligatory for skin tumor initiation in HPV8 transgenic mice treated with UV. These findings have important clinical implications for the development of skin cancer in humans.

## Results and discussion

**Fig. 1:**
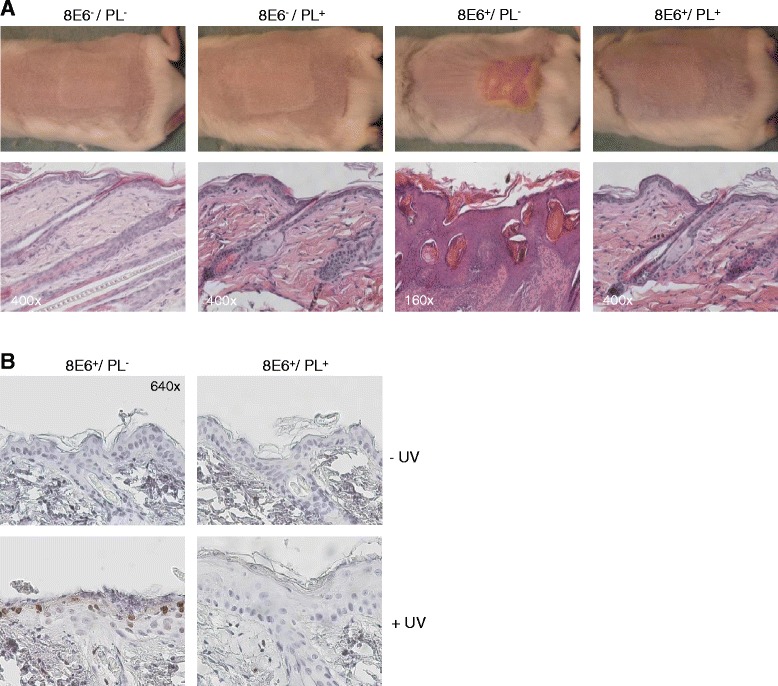
Repair of CPDs in K14-HPV8-E6wt mice abrogates UV-induced skin tumor formation. **a** K14-HPV8-E6wt animals (FVB/n background) were mated with K14-CPD-PL (FVB/n background) and offspring in F1 were irradiated with UV. The CPD-PL was reactivated by exposing animals to white fluorescent light tubes. Figure shows representative macroscopical (*upper panel*) and histological (*lower panel, magnifications are indicated*) skin images of E6^−^/PL^−^ (n = 11), E6^−^/PL^+^ (n = 11), E6^+^/PL^−^ (n = 11) and E6^+^/PL^+^ (n = 7) animals taken 24 days after UV-irradiation **b** Representative images of T^T stained skin sections of E6^+^/PL^−^ (n = 3) and E6^+^/PL^+^ (n = 3) mice collected 3 days after UV treatment and photoreactivation (magnification: 640×)

Enhanced repair of CPDs in K14-HPV8-E6wt mice abrogates UV-induced skin tumor formation. To delineate the physiological relevance of impaired DNA damage repair in skin tumor initiation by HPV8-E6 in vivo, the CPD-PL was expressed in HPV8-E6 cells by crossing K14-HPV8-E6wt with K14-CPD-PL animals. The resultant mice were irradiated with UV. E6^−^/PL^−^ and E6^−^/PL^+^ littermates were used as controls and did not develop any skin lesions. All E6^+^/PL^−^ animals developed UV-induced skin tumors three weeks after UVB treatment, in-line with previous findings that histologically showed papillomatosis and hyperkeratosis. However, reactivation of photolyase activity completely reversed the E6-induced skin phenotype resulting in a dramatic and complete suppression of tumor development in E6^+^/PL^+^ mice after UV irradiation (Fig. 1a). Staining for thymine dimers (T^T, as marker for CPDs) revealed that CPDs, undetectable in untreated skin, were present in UV treated E6^+^/PL^−^ skin but completely repaired 3 days after photoreactivation in double transgenic mice (Fig. 1b). Thus, elimination of CPDs by CPD-PL in K14 permissive skin cells of K14-HPV8-E6wt transgenic mice impaired initiation of papilloma growth after UV-irradiation. These results provide the first experimental in vivo evidence that the failure to repair UV-induced CPDs constitutes the initial step in HPV8-E6 mediated skin tumor development.

### HPV8-E6 interference with CPD repair is essential for papilloma formation

We have previously shown that expression of E6 of HPV5, closely related to HPV8, impairs the repair of UVB induced CPDs whereas E6 proteins of HPV10, 23, 24, 49 and 77 do not share this activity [17], suggesting that specific HPV types may confer a pre-disposition towards skin tumor development. We now show that in addition to HPV5 (*p* < 0.0001), HPV8 (*p* < 0.0001) and HPV20 (*p* = 0.0002) E6 proteins can also significantly delay the repair of UVB induced T^T (see Additional file 1 Figure S1), suggesting that these HPV types may present a greater tumorigenic risk.

**Fig. 2:**
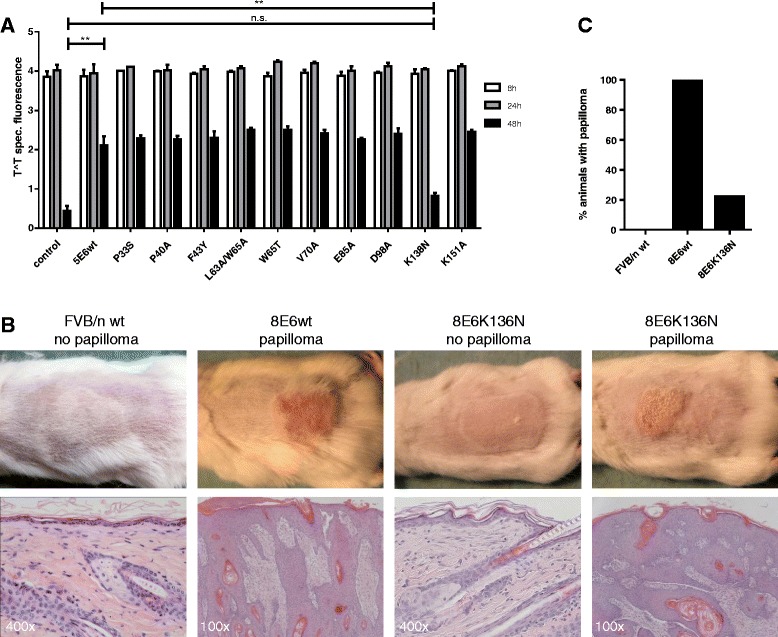
Inhibition of DNA damage repair by β-PV E6. **a** A panel of HPV5-E6 mutants were analysed using In-Cell Western to assay the delayed repair phenotype. The K138N mutant completely abrogated the ability of HPV5-E6 to delay the repair of UVB induced T^T (*n* = 3 in duplicate; control vs. HPV5-E6wt, **, *p* = 0.0032; HPV5-E6wt vs. HPV5-E6K138N, **, *p* = 0.0057; control vs. HPV5-E6-K138N, *p* = 0.07). Data are presented as mean ± SEM. **b** Representative macroscopical images (*upper panel*) and histology (lower panel, magnification as indicated) of FVB/n-wt (*n* = 15), K14-HPV8-E6wt (*n* = 12) and K14-HPV8-E6K136N animals (*n* = 50) taken 24 days after UV-irradiation. **c** Bar diagram showing percentage of animals developing papillomas after UV-irradiation

In order to characterize further the activity of E6 involved in the interference with DNA repair, a panel of cell lines expressing previously characterized E6 proteins with point mutations at residues conserved in HPV5 and HPV8 E6 were investigated. All mutant proteins were found to be stably expressed and retained the ability to inhibit UVB-induced apoptosis [18]. Of the E6 mutants tested, only K138N was severely impaired in its ability to interfere with T^T repair (K138N versus E6wt, *p* = 0.0057; K138N versus control, *p* = 0.07; student t-test), while all other mutants showed an activity that was similar to that of the wild-type HPV5-E6 protein (HPV5-E6wt versus control, *p* = 0.0032, student t-test). This data suggest that the integrity of the residue K138 is important for E6 to delay T^T repair (Fig. 2a), and that the inhibition of apoptosis and interference with DNA repair pathways are functionally separate activities of E6.

We then generated a K14-HPV8-E6K136N transgenic mouse model (K136 in HPV8-E6 corresponds to HPV5-E6-K138) to examine whether UV-induced skin tumor development is impaired in these animals. Five weeks old animals without skin abnormalities were irradiated once with an inflammatory radiation dose known to cause ‘sunburn’ (apoptotic) cell formation. After UV treatment none of the mice lacking E6 expression had developed skin lesions and their skin healed completely from the UVB induced hyperplasia. While all K14-HPV8-E6wt mice developed papillomas within 3 weeks post irradiation, only 22 % of K14-HPV8-E6K136N mice showed skin tumor formation, but in 78 % of E6 mutant mice the skin had healed completely (Fig. 2 b,c). Tumor formation in the E6 mutant mice may be due to the residual activity of K136 on the repair of T^T. Since the mRNA levels of E6 play an important role in papilloma induction after UV treatment, we compared the E6 expression levels in K14-HPV8-E6wt and K14-HPV8-E6K136N mouse lines. E6 mRNA was measured by qRT-PCR in RNA from shaved skin biopsies. Similar E6 levels were found in untreated normal skin (*p* = 0.5414). At day 3 after UV irradiation, the E6 levels increased to a similar extent in both lines and showed no significant difference (*p* = 0.2904), indicating that differences in E6 expression levels are not responsible for observed mouse skin phenotype (see Additional file 2: Figure S2). These findings show that the ability of E6 to interfere with T^T repair is critical for skin tumor formation following UV exposure.

### DNA damage persists in UVB irradiated skin expressing HPV8 E6

**Fig. 3:**
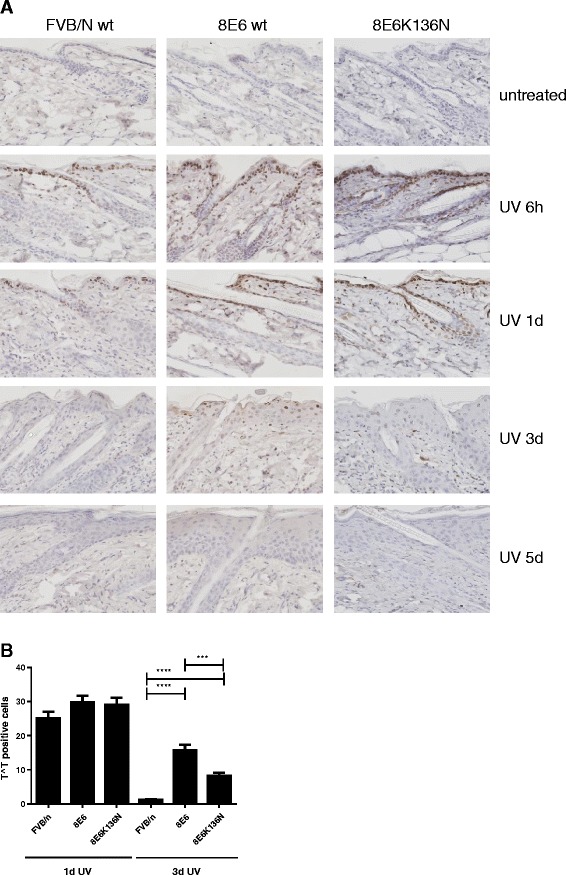
Persistence of DNA damage in UVB treated skin of K14-HPV8-E6 mice. **a** Paraffin embedded skin sections of UV treated skin from FVB/n-wt, K14-HPV8-E6wt and K14-HPV8-E6K136N mice were stained for T^T (magnification: 400×). Representative images of *n* = 4 skin biopsies per time-point per mouse line are shown. **b** T^T positive cells were quantified by counting positive cells per 3 fields of *n* = 4 animals per mouse line and time-point. At day 1 after UV treatment, no significant difference in the number of T^T positive cells were observed (FVB/n-wt vs. K14-HPV8-E6wt, *p* = 0.1094; FVB/n-wt vs. K14-HPV8-E6-K136N, *p* = 0.1769; K14-HPV8-E6wt vs. K14-HPV8-E6-K136N, *p* = 0.8115). Three days after treatment significantly more T^T positive cells persisted in the skin of K14-HPV8-E6wt mice (FVB/n-wt vs. K14-HPV8-E6wt, ****, *p* < 0.0001; FVB/n-wt vs. K14-HPV8-E6-K136N, ****, *p* < 0.0001; K14-HPV8-E6wt vs. K14-HPV8-E6-K136N, ***, *p* = 0.0003). Data are presented as mean ± SEM

Having found that E6 expression interfered with T^T repair and that enhanced T^T repair abrogated the tumorigenic potential of E6, we next asked whether CPD lesions persisted and could be detected in UV treated skin of these animals by immunohistochemistry. As expected, T^Ts were not detected in non-irradiated skin of any of the animals examined, however T^Ts were readily detected 6 h after UV irradiation. One day after UV treatment no difference in T^T levels were detected between all mouse lines. However, three days after UV irradiation only few T^T positive cells could be detected in FVB/n-wt skin indicating efficient repair of these lesions. Significantly more T^T positive cells persisted in the skin of K14-HPV8-E6wt compared to FVB/n-wt control (*p* < 0.0001) and K14-HPV8-E6-K136N mutant mice (*p* = 0.0003) (Fig. 3a,b).

**Fig. 4:**
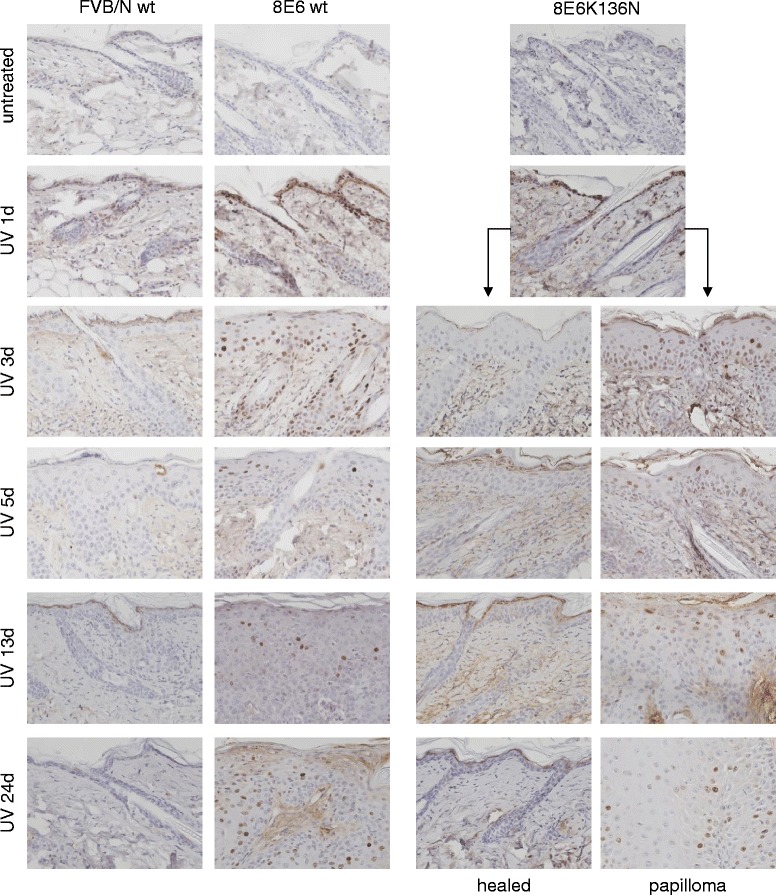
Presence of DNA damage in UV treated skin of K14-HPV8-E6 mice. Paraffin embedded skin sections of UV treated skin were stained for γH2AX (magnification: 400×). Representative images of *n* = 4 mouse skin biopsies per time-point are shown

Expression of HPV E6 proteins can bypass the G1 to S phase cell cycle checkpoint [17]. As unrepaired T^T can lead to the generation of highly genotoxic and potentially mutagenic DSBs by DNA replication fork collapse during S-phase, we also analyzed the skin of these three mouse lines for the presence of phosphorylation of the histone variant H2AX (termed γH2AX) that is indicative of the presence of DSBs. Three days after UVB-treatment γH2AX was not detected in cells of FVB/n-wt mice, while in K14-HPV8-E6wt mice γH2AX was found at both early (3d, 5d) and later (13d, 24d) time points when papillomas had formed (Fig. 4). About 80 % of skin biopsies of K14-HPV8-E6K136N mice collected at 3, 5, 13 and 24 days after treatment showed a γH2AX staining intensity comparable to FVB/n-wt, while about 20 % showed a staining pattern similar to K14-HPV8-E6wt, a staining pattern that was comparable to the tumor rate found in these animals. These results indicate that the ability of E6 to maintain DNA damage together with its ability to over-ride normal cell cycle checkpoints, thereby allowing damaged cells to persist and replicate even whilst harboring DNA lesions, leads to the generation of highly mutagenic lesions that are known to be associated with tumor formation.

### E6-impairment of DNA damage repair is associated with inhibition of DNA damage sensing

**Fig. 5:**
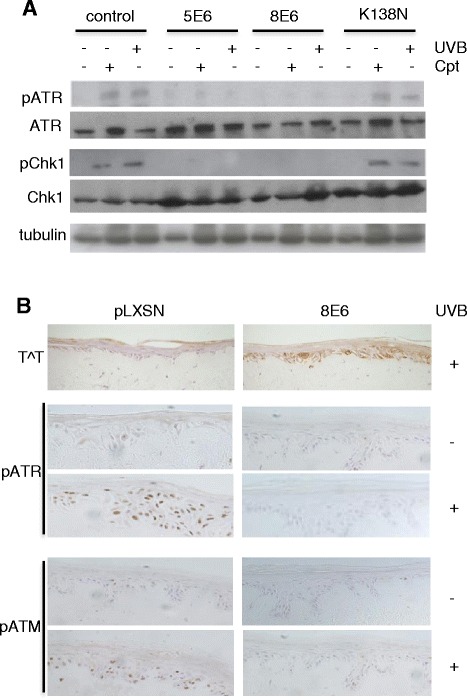
Expression of E6 inhibits phosphorylation of ATM and ATR. **a** Representative immunoblots (*n* = 3) showing that HPV5 and HPV8 E6 inhibit ATR as well as Chk1 phosphorylation measured 24 h following UVB irradiation or 4 h of camptothecin treatment. Normal phosphorylation patterns are restored through mutation of HPV5-E6 at K138. Total levels of ATR and Chk1 were unaffected by E6 expression. **b** Expression of HPV8-E6 in organotypic cultures delays DNA repair and DNA damage sensing. Cultures expressing HPV8-E6 or pLXSN empty vector grown in parallel were irradiated with 20 mJ/cm^2^ UVB 24 hours prior to fixation. Representative immunohistochemical staining (*n* = 3) experiments showing phosphorylation of ATM and ATR in control cells and lack of phosphorylation in HPV8-E6 expressing cells, which correlates with the presence of un-repaired T^∧^T

A key checkpoint in precancerous lesions that acts as a barrier to constrain tumor cell progression is the activation of the DNA damage response (DDR) [19, 20], that involves activation of two kinases, ATM and ATR, together with their down stream effectors Chk1 and Chk2 that regulate multiple proteins involved in cell cycle control and apoptosis. Indeed, ATM appears to be the main kinase phosphorylating H2AX in response to random DNA DSBs, whereas ATR phosphorylation of H2AX is associated with UVC damage or replication stress [21]. To study the impact of E6 on DDR mediated sensing of DNA damage early after UVB treatment, cell lines expressing HPV5-E6wt, HPV5-E6K138N or HPV8-E6wt were generated and treated with camptothecin (CPT, a DNA damaging agent that generates DSBs), or irradiated with 5 mJ/cm^2^ UVB. While control cells lacking E6 expression mounted a DDR as evidenced by phosphorylation of ATR (Ser428), expression of either HPV5 or HPV8 E6 protein blocked ATR phosphorylation (Fig. 5a). The detection of low levels of pATR in the E6 cells was however not due to E6-induced proteolysis as total levels of ATR were not significantly affected. The phosphorylation pattern for Chk1 (Ser317) correlated with the activation of ATR, providing further evidence that this signaling pathway is inhibited by expression of E6. In contrast, expression of the HPV5-E6K138N mutant that was impaired for inhibition of T^T repair did not alter the phosphorylation patterns of either ATR or Chk1.

The activation of the DDR was also investigated in organotypic skin cultures generated from primary human adult keratinocytes that had been transduced with an E6-expressing retroviral construct or empty vector. Immunohistochemical analysis revealed the presence of T^T in UVB-treated E6 cultures 24 h post UVB treatment, whereas T^T were not detected in the UVB-treated control culture (Fig. 5b). Thus, the pattern of T^T staining in epidermal layers of primary keratinocytes correlated closely with the detection of T^T in cell lines. While phosphorylated ATR and ATM was observed in the nuclei of control cultures lacking E6 expression, in HPV8-E6 cultures no ATM or ATR phosphorylation was found, a staining pattern that correlated inversely with the detection of T^T following UVB irradiation. These findings show that while DNA damage persists in E6-expressing cells they fail to sense and mount a DDR, thereby by-passing a critical barrier to tumor formation.

### Impaired p300 binding by HPV8-E6K136N

**Fig. 6:**
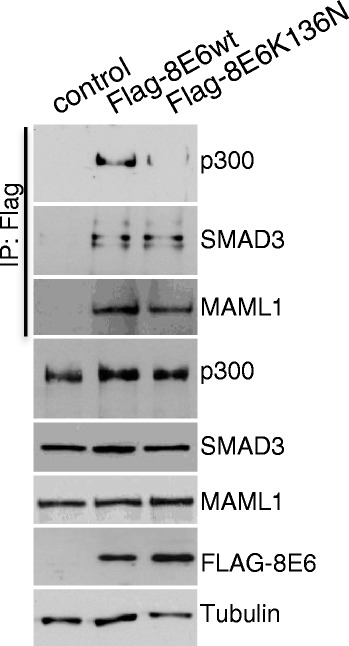
Impaired p300 binding by HPV8-E6K136N. Extracts from RTS3b cells, which were transiently transfected with expression vectors for the empty vector, Flag-8E6wt or Flag-8E6K136N were incubated with M2-FLAG-agarose. Co-immunoprecipitated p300, MAML1 and SMAD3 and 10 % of the input extracts were detected by Western blot with specific antibodies. The expression of HPV8-E6 was confirmed by a Western blot against the Flag tag. Equal protein loading was confirmed by tubulin expression

The interference of cutaneous E6 proteins with p300 is a property that is needed for cellular immortalization and tumorigenesis and E6 mutant proteins that have lost the ability to bind p300 cannot execute this tumorigenic activity (Münch et al., 2010). It was previously shown that HPV8-E6 interacts with the cellular transcription co-activator and histone acetyl-transferase p300 [22–27] and that a deletion mutant of HPV8-E6 protein lacking amino acid 132–136 does not bind p300 anymore [22]. Since K136 lies within the p300-binding domain of HPV8-E6 we analysed whether HPV8-E6K136N still interacts with p300 in keratinocytes. As shown in Fig. 5c, levels of p300 in total cell extracts were not changed upon HPV8-E6 expression. While HPV8-E6wt bound p300, the mutant HPV8-E6K136N nearly completely lost the ability to complex with p300. To exclude that missing binding to p300 resulted from a changed tertiary structure of HPV8-E6K136N, the ability of the mutant protein to bind to the known cellular target proteins MAML1 and SMAD3 [25] was studied. The aa substitution of K136N in HPV8-E6 did not affect binding to MAML1 and SMAD3 (Fig. 6). At least in RTS3b keratinocytes and C33a cells (data not shown) we did not observe degradation of p300 upon HPV8-E6 expression in contrast to findings of Howie et al. (2011) [24] in other cell types. In line with stable p300 levels, total amounts of ATR were also not significantly affected (Fig. 5a), whereas Wallace et al. (2012) [28] observed reduced ATR levels correlating with p300 degradation. In summary, our data suggest that HPV8-E6 binding to p300 correlates with reduced levels of phosphorylated ATR and impaired DDR without affecting the levels of p300 and total ATR.

## Conclusion

Since β-PV are part of the normal microbiological flora of the skin, viral infection per se does not represent the major event in skin carcinogenesis [29]. The high prevalence of β-PV infection in healthy humans and low levels of viral DNA in the skin is raising the question how β-PV could affect SCC development in humans. Given that β-PV DNA loads in premalignant lesions exceed those in SCC, the interaction of β-PV E6 oncoprotein with the DNA repair mechanisms at early stage of skin tumor formation may allow non-repaired or incorrectly repaired UV-induced lesions to persist, and this, coupled with the anti-apoptotic activity of E6, can facilitate the generation and propagation of deleterious mutations that drive tumor initiation and progression. Our results provide the first experimental in vivo evidence that impairment of the DNA repair machinery in basal cells is necessary for initiation of papilloma growth by E6 and that CPD lesions are mandatory for E6-mediated tumorigenesis. The interference of E6wt with phosphorylation of the cell cycle checkpoint kinases and p300 may have contributed to accumulation of DSBs and to the relief of cell cycle arrest resulting in skin hyperplasia in K14-HPV8-E6wt mouse. Our findings provide the first in vivo mechanistic data on the tumorigenicity of HPV8 and direct evidence supporting the hypothesis that β-PV may play a role early in skin cancer development by enhancing the genotoxic effects of UV light.

## Materials and methods

### Plasmids

For generation of HA-tagged HPV5, HPV8 and HPV20 E6 proteins, corresponding ORFs were amplified by PCR from viral genomes with specific primers flanking a BamHI and XhoI restriction sites. The PCR products were then ligated into pcDNA3.1-5’HA [17]. The pcDNA3.1-5’HA based mutants of HPV5-E6 have been described previously [18]. Site-directed mutagenesis of HPV8-E6 in K14CreERtam-HPV8-E6 (also called K14-HPV8-E6; [12]) and pcDNA3.1-Flag-HPV8-E6 was performed using the QuickChange Site-directed mutagenesis kit (Stratagene) with the primers HPV8-E6K136N-fw: 5’ CGTCCCTTTCATAACGTTAGAGGAGGCTG 3’ and HPV8-E6K136N-bw: 5’ CAGCCTCCTCTAACGTTATGAAAGGGACG 3’, leading to an AAA → AAC exchange giving rise to HPV8-E6K136N.

### Commercial antibodies

The antibodies used in this study were the following: anti-thymine dimer (Insight Biotechnology), (anti-γH2AX, MABE205, Millipore, Schwalbach, Germany), anti-pATM (ser1981, Rockland), anti-ATR (Santa Cruz), anti-pATR (ser428, Cell Signalling), anti-Chk1 (Santa Cruz), anti-pChk1 (ser317, Cell Signalling), and anti-tubulin (YL1/2, Abcam), FLAG-M5 monoclonal antibody (A2220, Sigma), anti-p300 (C-20, Santa Cruz), anti-tubulin (YL1/2, Abcam), anti-MAML1 (Cell Signalling), anti-SMAD3 (Abcam).

### Cell culture, transfection and western blot

HT1080 cells were maintained in DMEM plus 10 % foetal calf serum supplemented with antibiotics, in a humidified atmosphere at 37 °C, 5 % CO^2^. Polyclonal cell lines were generated by transfecting plasmid DNA using FuGENE 6 transfection reagent (Roche) according to the manufacturer’s instructions. Exponentially growing cells were treated with 6 μM camptothecin (CPT, Sigma) for 4 h prior to protein harvesting or irradiated with 5 mJ/cm2 UVB, using a UV Products CL400 cross-linker fitted with F8T5 bulbs that give a sharp emission peak at 312 nm, and cultured for a further 8, 24 or 48 h. T^T in HT1080 cells were quantified using an In-Cell Western methodology employing the LiCOR Odyssey immunofluorescence detection system. For this, cells were then washed twice in PBS and fixed in 3.7 % paraformaldehyde for 20 min at RT and then permeablized using 0.1 % TritonX-100 in PBS for 10 min. Cells were blocked in Odyssey Blocking Buffer (OBB, LiCOR) diluted 1:1 in PBS for 1 h at RT. T^T were detected using the anti-thymine dimer antibody at 1:500 dilution in OBB/PBS plus 0.1 % Tween20 followed by sheep anti-mouse IRDye™ 800CW at 1:800 dilution (Rockland Immunochemicals) in OBB plus 0.2 % Tween-20. Syto60 nucleic acid stain (LiCOR) was used at a 1:5000 dilution in OBB/PBS/Tween for normalization of the dimer signal. Cells were visualized and fluorescence quantified using a LiCOR Odyssey Infrared Imaging Scanner and quantification software. For Western blots with whole cell protein extracts adherent cells were lysed in RIPA buffer. 20 μg of protein was loaded onto SDS-PAGE gels and transferred onto nitrocellulose membrane according to standard procedures. The human keratinocyte cell line RTS3b was maintained in RM+ media [30]. 2,5 × 10^5^ cells were seeded in 10 cm dishes and transfected with 10 μg of plasmid DNA with FuGENE 6. Two days after transfection the cells were washed with PBS and harvested by scraping in 100 mM LSDB buffer (100 mM KCL, 50 mM Tris–HCl, 20 % glycerol and 0.1 % NP-40, 1 mM dithiothreitol, 1 mM phenylmethylsulfonyl fluoride and 1× protease inhibitor Complete. For Co-IP experiments, extracts were incubated with FLAG-M2 antibody coupled to agarose (A2220, Sigma) for 2 h at 4 °C, followed by three washes with LSDB containing different KCl concentrations. Cellular proteins binding to E6 were detected by Western blotting with specific antibodies. De-epidermalized human dermis based organotypic cultures of primary human keratinocytes expressing HPV8-E6 were previously described [31, 32].

### Mouse lines

Mouse lines used in this study included FVB/n-wt (Charles River Laboratories, Sulzfeld, Germany), the transgenic hemizygous FVB/N line K14-HPV8-E6wt [12] and the transgenic hemizygous C57BL/6 J mice expressing the *Potorous tridactylus* CPD photolyase (CPD-PL) under the control of the hK14 promoter (K14-CPD-PL; [5, 7]). To generate the K14-HPV8-E6K136N line, the linearized transgene, in which the HPV8-E6K136N gene is under the control of the human keratin-14 (K14) promoter, was microinjected into the pro-nucleus of fertilized FVB/n oocytes, which were implanted into pseudopregnant surrogate mothers to produce putative founder mice. To detect transgenic mice PCR analysis was performed as described previously [12]. Briefly, genomic DNA was isolated from tail biopsies of 3-week-old mice using the QIAmp Tissue kit (Qiagen, Hilden, Germany). Samples of genomic mouse DNA were analysed for presence of the transgene by PCR, using the following primers: HPV8-E6-fw: ggatcctttcctaagcaaatggacggg; HPV8-E6-bw: ggatccgcatgccacaaaatcttgcacagtgacctc; CPD-PL-fw: tgagactcatctcccaggac; CPD-PL-bw: caccaatgccatgtgtttgc. The PCR reaction conditions consisted of a 3-minute denaturation step (95 °C) and 35 cycles of amplification (95 °C, 30 seconds; 60 °C, 1.5 min; 72 °C, 1 min). K14-CPD-PL mice (C57/BL6) were back-crossed with FVB/n-wt animals for 5 generations and then mated to K14-HPV8-E6wt (FVB/n).

### UV irradiation and photoreactivation of mouse skin

UV irradiation protocols were approved by the governmental animal care office North-Rhine-Westphalia (Leibnizstraße 10, 45659 Recklinghausen, protocol no. 8.87–50.10.35.08.163) and were in accordance with the German Animal Welfare Act as well as the German Regulation for the protection of animals used for experimental purposes. For dorsal caudal skin irradiation age (5 weeks) and sex matched mice were irradiated once with 10 J/cm^2^ UVA and 1 J/cm^2^ UVB on a 4 cm^2^ sized area. For photoreactivation after UV treatment, double transgene positive animals were exposed to the light of 4 white fluorescent tubes (GE Lightning Polylux XL F36W/840) filtered through 5 mm of glass. All offspring were macroscopically examined for the presence of skin lesions on day 34 after UV treatment.

### Immunohistochemistry

4 μM sections on polylysine coated slides from formalin fixed, paraffin-embedded organotypic cultures and mouse skin were analysed. Sections were deparaffinised by washing in 100 % Xylene, rehydrated through washing in decreasing concentrations of ethanol. Sections were then incubated in 3 % hydrogen peroxide in methanol for 20 min to inhibit endogenous peroxidises. Antigen unmasking was performed by boiling the tissue sections in 10 mM citric buffer for 3 min in a beaker in a microwave followed by 15 min resting at RT. Sections were then blocked in 50 % horse serum in PBS (v/v) for 30 min. Primary antibody was diluted in 2 % horse serum/PBS and incubated overnight at 4 °C. A biotinylated secondary antibody was applied and slides were visualized using a streptavidin-biotin-peroxidase detection system (Vectastain ABC or M.O.M. kit, Linaris, Dossenheim, Germany) using DAB (3,3’-diaminobenzidine) liquid substrate (Biogenex, Fremont, CA, USA). Sections were counterstained in Gills Haematoxylin and dehydrated through washing in increasing concentrations of ethanol, then mounted in DePeX mounting medium (Serva, Heidelberg, Germany) and visualised using an Zeiss Axiophot microscope and imaging software.

### qRT-PCR

Total RNA isolation, reverse transcription and qPCR were performed as described previously [33]. Total RNA was isolated from tissues and cells using the RNeasy Kit and DNAse digestion was performed on column using RNAse-free DNAse according to the manufacturer’s instructions (Qiagen, Hilden, Germany). One μg of total RNA was reverse transcribed using the Omniscript RT Kit (Qiagen, Hilden, Germany) with 10 μM Random nonamers (TIB MOLBIOL, Berlin, Germany) and 1 μM oligo-dT23-primer (Sigma, Deisenhofen, Germany) as well as 10 units RNAse Inhibitor (Fermentas, St. Leon-Rot, Germany). QPCR was performed using the Light-Cycler System (Roche, Mannheim, Germany). Total transcript numbers of the target gene were normalized to the total copy number of the house-keeping gene hypoxanthine phosphoribosyltransferase 1 (HPRT1). One PCR reaction contained 2 μl of 1:10 diluted cDNA in a total volume of 20 μl, 1.25 units Platinum Taq Polymerase and the provided buffer (Invitrogen, Karlsruhe, Germany), 4 mM MgCl2, 1.6 μl of a 1:1000 dilution of SYBGreen (Sigma, Deisenhofen, Germany), 5 % DMSO, 0.5 μM of forward and backward primer each, 500 ng/μl non-acetylated bovine serum albumin (Fermentas, St. Leon-Rot, Germany) and 0.2 mM deoxynucleotide triphosphates each. Amplified PCR fragments were cloned into pJET1.2 (Qiagen, Hilden, Germany) to generate absolute standards with primers also used for subsequent qPCR analysis. Samples were analysed in duplicate together with a 10-fold dilution series of standard plasmid. The cycling protocol conditions were 10 minutes at 95 °C, followed by 40 cycles of 15 second at 95 °C (20 °C/s), 30 seconds at 55 °C (20 °C/s) and 30 seconds at 72 °C (20 °C/s). The primers used in this study were the following: mHPRT1-fw: cctaagatgagcgcaagttgaa; mHPRT1-bw: ccacaggactagaacacctgctaa; HPV8-E6-fw: ccgcaacgtttgaatttaatg; HPV8-E6-bw: attgaacgtcctgtagctaattca.

### Statistical analysis

All experiments were repeated a minimum of three times. All data from In Cell Western blot assays and qRT-PCRs were expressed as mean ± SEM. The data presented as immunoblots or images of immunohistochemical analysis are from a representative experiment, which was qualitatively similar in the replicate experiments. Statistical significance was determined with unpaired 2-tailed Student’s *t*-test. The asterisks shown in the figures indicate significant differences of experimental groups (*p < 0.05; **p < 0.01, ***p < 0.001, ****p < 0.0001).

## Additional files

Additional file 1: Figure S1. Inhibition of T^T repair by β-PV E6. HT1080 cells expressing E6 genes of β-PV types 5, 8 and 20 were irradiated with UVB and levels of T^∧^T were assayed using In-Cell Western analysis (*n* = 4 in duplicate, HPV5,****, *p* < 0.0001; HPV8, ****, *p* < 0.0001; HPV20, ***, *p* = 0.0002). Data are presented as mean ± SEM. (PPT 151 kb) Additional file 2: Figure S2. Comparable E6 mRNA expression levels in mouse skin. Skin biopsies from K14-HPV8-E6wt and K14-HPV8-E6K136N lines were taken at the indicated time points after UV irradiation and HPV8 E6 mRNA levels were measured in duplicate by qRT-PCR and normalized to the mRNA levels of HPRT1 (*n* = 6; untreated skin, *p* = 0.5414; 3d post UV-treatment, *p* = 0.2904). Data are presented as mean ± SEM. (PPTX 59 kb)

## Abbreviations

PV: Papillomavirus
T^T: Thymine dimer
CPD: Cyclobutane-pyrimidine-dimer
γH2AX: Phosphorylated form of histone variant H2AX
PL: Photolyase
ATM: Ataxia Telangiectasia Mutated
ATR: Ataxia Telangiectasia And Rad3 Related
Chk1: Checkpoint Kinase 1
