# Supplementary figures and images for: Human papillomavirus mediated inhibition of DNA damage sensing and repair drives skin carcinogenesis
Source: Mol Cancer. 2015 Oct 29;14:183. doi: 10.1186/s12943-015-0453-7 (PMC4625724; doi:10.1186/s12943-015-0453-7)

## Slide 1
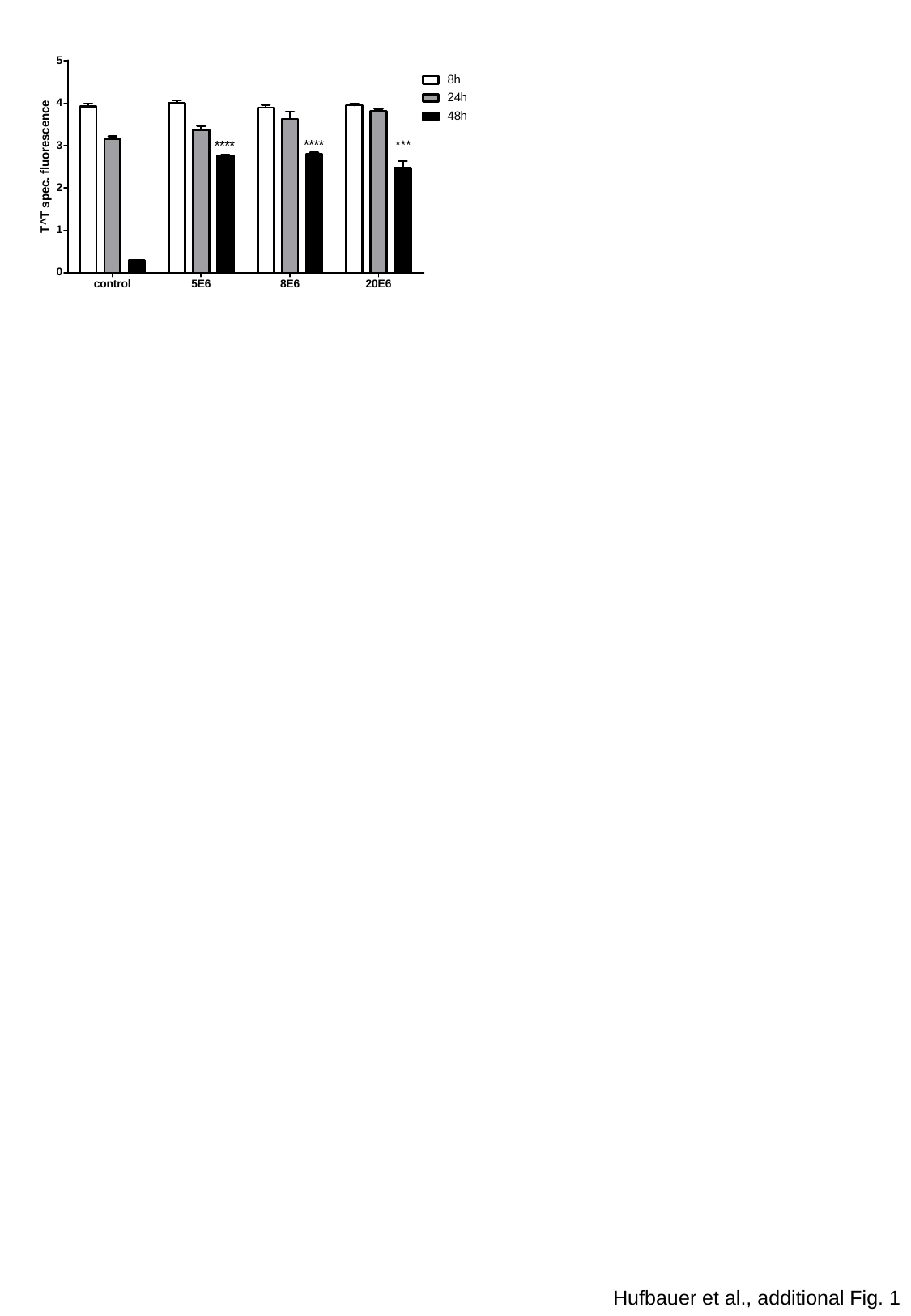

Hufbauer et al., additional Fig. 1

Supplement: Additional file 1: Figure S1. — Inhibition of T^T repair by β-PV E6. HT1080 cells expressing E6 genes of β-PV types 5, 8 and 20 were irradiated with UVB and levels of T∧T were assayed using In-Cell Western analysis (n = 4 in duplicate, HPV5,****, p < 0.0001; HPV8, ****, p < 0.0001; HPV20, ***, p = 0.0002). Data are presented as mean ± SEM. (PPT 151 kb) [file 12943_2015_453_MOESM1_ESM.ppt]

## Slide 1
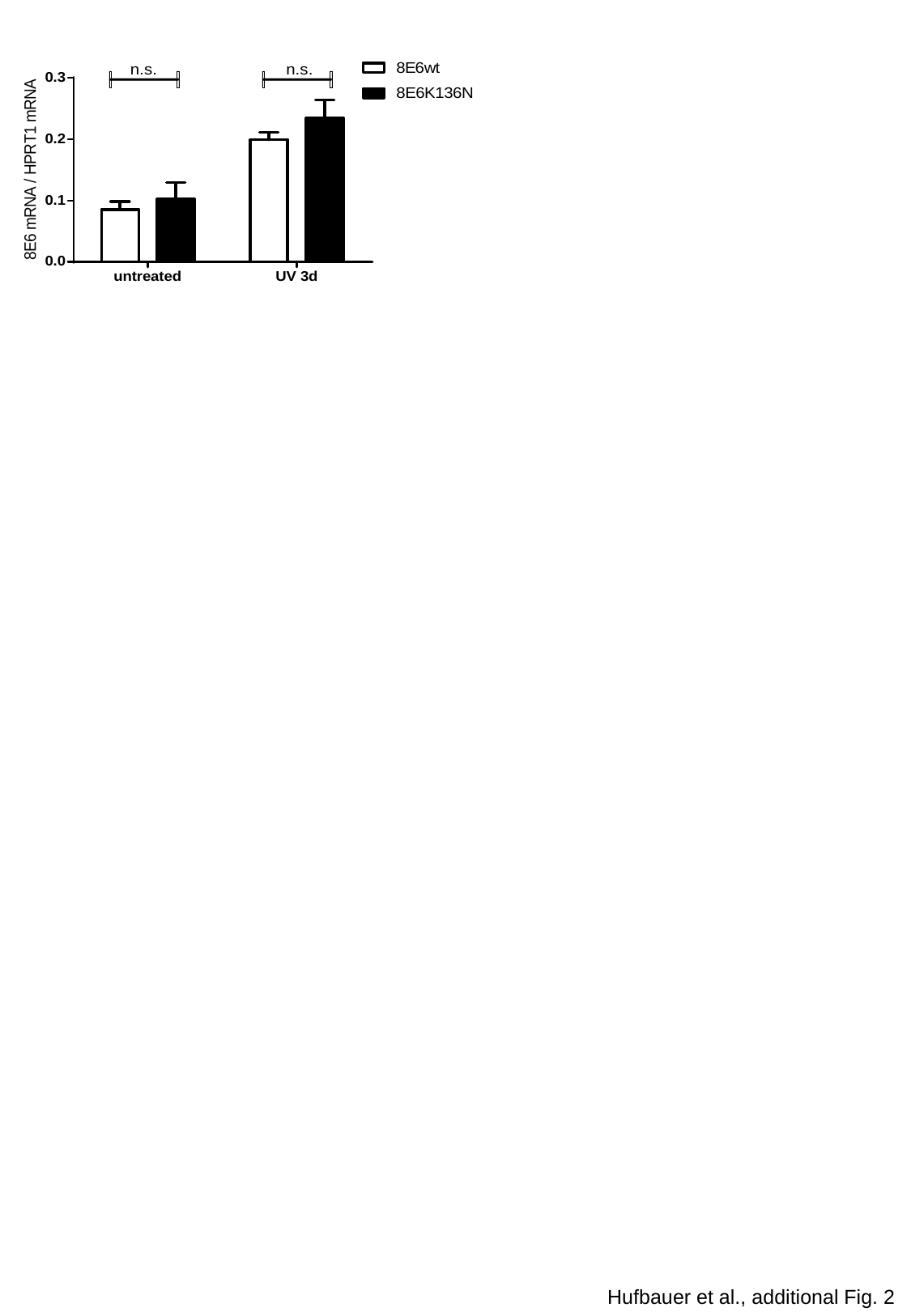

Hufbauer et al., additional Fig. 2

Supplement: Additional file 2: Figure S2. — Comparable E6 mRNA expression levels in mouse skin. Skin biopsies from K14-HPV8-E6wt and K14-HPV8-E6K136N lines were taken at the indicated time points after UV irradiation and HPV8 E6 mRNA levels were measured in duplicate by qRT-PCR and normalized to the mRNA levels of HPRT1 (n = 6; untreated skin, p = 0.5414; 3d post UV-treatment, p = 0.2904). Data are presented as mean ± SEM. (PPTX 59 kb) [file 12943_2015_453_MOESM2_ESM.pptx]
